# Design of lipid nanoparticle delivery agents for multivalent display of recombinant Env trimers in HIV vaccination

**DOI:** 10.1186/1742-4690-9-S2-O10

**Published:** 2012-09-13

**Authors:** S Pejawar-Gaddy, J Kovacs, D Barouch, B Chen, D Irvine

**Affiliations:** 1 Massachusetts Institute of Technology, Boston, MA, USA; 2 Harvard University School of Medicine, Boston, USA

## Background

Immunization strategies that elicit antibodies capable of neutralizing diverse strains of the virus will likely be an important part of a successful vaccine against HIV. The envelope trimer is the only neutralizing target on the virus, and strategies to promote durable, high avidity antibody responses against the native intact trimer structure are lacking. We recently developed chemically-crosslinked lipid nanocapsules as carriers of molecular adjuvants and encapsulated or surface-displayed antigens, which promote follicular helper T-cell responses and elicited high-avidity, durable antibody responses to a candidate malaria antigen (Moon et al. Nat. Mater. 10 243 (2011); Moon et al. PNAS 109 1080 (2012)).

## Methods

To apply this system to the delivery of HIV antigens, we developed a strategy to anchor recombinant envelope trimers to the surfaces of these particles under conditions preserving the antigenic integrity of the trimers, allowing multivalent display of these immunogens for immunization. To anchor trimers in their native orientation, gp140 trimers with terminal his-tags were anchored to the surface of lipid nanocapsules via Ni-NTA-functionalized lipids.

## Results

Owing to their significant size (409 kDa) and heavy glycosylation, we found that liquid-ordered and/or gel-phase lipid compositions were required to stably anchor trimers to particle membranes. Trimer-loaded nanocapsules carrying monophosphoryl lipid A elicited durable antibody responses with titers comparable to a Complete Freund’s Adjuvant (CFA)-like emulsion in mice, without the toxic inflammation associated with the latter adjuvant. Further, nanocapsules elicited strong helper T-cell responses associated with a steadily increasing avidity of trimer-binding antibody over 90 days, which was not replicated by other adjuvants.

## Conclusion

These results suggest that nanoparticles displaying HIV trimers in an oriented, multivalent presentation can promote key aspects of the humoral response against Env immunogens.

This work was funded by the NIH (AI095109) and the Ragon Institute of MGH, MIT, and Harvard.

